# Novel imaging diagnosis of neuropsychiatric systemic lupus erythematosus using topological data analysis: A retrospective study

**DOI:** 10.1371/journal.pone.0329859

**Published:** 2025-08-13

**Authors:** Kayoko Urashima, Kunihiro Ichinose, Hideki Ishimaru, Hirokazu Kumazaki, Atsushi Kawakami, Masao Ueki

**Affiliations:** 1 Department of Neuropsychiatry, Nagasaki University Graduate School of Biomedical Sciences, Nagasaki, Japan; 2 Department of Immunology and Rheumatology, Division of Advanced Preventive Medical Sciences, Nagasaki University Graduate School of Biomedical Sciences, Nagasaki, Japan; 3 Department of Rheumatology, Shimane University Faculty of Medicine, Izumo, Japan; 4 Department of Radiological Sciences, Nagasaki University Graduate School of Biomedical Sciences, Nagasaki, Japan; 5 School of Information and Data Sciences, Nagasaki University, Nagasaki, Japan; Transilvania University of Brasov: Universitatea Transilvania din Brasov, ROMANIA

## Abstract

**Introduction:**

Diagnosing neuropsychiatric systemic lupus erythematosus (NPSLE) and differentiating it from systemic lupus erythematosus (SLE) without neuropsychiatric manifestations remains a substantial clinical challenge due to the absence of specific biomarkers. Topological data analysis (TDA) is a novel computational technique that enables the visualization, exploration, and analysis of complex data structures. This study aimed to identify distinct neuroimaging biomarkers in patients with NPSLE (NPSLE group) and differentiate them from patients with SLE without neuropsychiatric symptoms (non-NPSLE group) by employing TDA.

**Methods:**

We conducted a retrospective cohort study involving 30 patients with NPSLE and 30 without neuropsychiatric symptoms between 2005 and 2020. TDA was utilized to extract topological features, specifically connected components and holes, from fluid-attenuated inversion recovery (FLAIR) sequences obtained via brain magnetic resonance imaging (MRI). Summary statistics, including critical point count, persistence lifetime, centroid coordinates, perimeter, area, and filamentarity, were derived from persistence diagrams.

**Results:**

Multiple logistic regression analyses, adjusted for age, cerebrovascular comorbidities, and 50% hemolytic unit of complement levels, demonstrated a significant association between NPSLE and the perimeter of the holes (odds ratio [OR]: 1.67, 95% confidence interval [CI]: 1.07–2.63, **p* *= 0.025) and the area of the holes (OR: 4.42, 95% CI: 1.35–19.6, **p* *= 0.026) of the identified topological features. Additionally, both areas under the receiver operating characteristic curve (AUC) exceeded 0.8, indicating good diagnostic accuracy.

**Conclusion:**

This study identified novel neuroimaging biomarkers for the diagnosis of NPSLE. The application of TDA to brain MRI features in patients with SLE proved to be a valuable diagnostic tool, particularly through the analysis of persistence diagrams.

## Introduction

Systemic lupus erythematosus (SLE) is a chronic autoimmune disease that can affect multiple organ systems, including the skin, joints, kidneys, and central nervous system [1]. It predominantly affects young women of childbearing age. Neuropsychiatric SLE (NPSLE) is associated with a wide array of neurological and psychiatric manifestations, including peripheral neuropathy, stroke, headaches, seizures, cognitive dysfunction, depression, and psychosis [2–4]. Neuropsychiatric symptoms manifest in 15%–95% of patients with SLE [4–7] and recur in 17.4% of cases [6]. Recurrent neuropsychiatric events are associated with adverse long-term outcomes, including a 7%–19% increase in mortality [8]. Therefore, neuropsychiatric complications of SLE significantly affect patient quality of life and long-term prognosis [9].

Sarbu et al. documented that brain magnetic resonance imaging (MRI) in patients with NPSLE often reveals abnormalities such as focal white matter hyperintensities (WMHs) and cerebral atrophy [10]. However, brain MRI findings are unremarkable in over 50% of patients with NPSLE, and WMHs on T2-weighted imaging are observed in 20%–50% of patients with SLE, irrespective of neuropsychiatric involvement, as well as in 47% of healthy individuals [11]. Although abnormal MRI findings are more prevalent in patients with CNS-involved SLE than in those without, the positive predictive value remains 42%, and the negative predictive value is 76%, which is inadequate for diagnostic purposes [12]. WMHs and cerebral atrophy are frequently observed in patients with SLE but are not specific to NPSLE [13]. Despite indications of structural abnormalities in NPSLE, the application of basic metrics such as global atrophy and WMH lesion count does not result in an NPSLE diagnosis [14,15].

Recent advancements in artificial intelligence and machine learning for biomedical data analysis offer new avenues for descriptive, predictive, and prescriptive analyses that surpass the capabilities of manual methods [16–18].

Topological data analysis (TDA) is an emerging data science technique that has garnered attention for its utility in quantifying topological features [19–22]. TDA is a method that analyzes the birth and death of topological features—such as connected components and holes—by gradually increasing the radius around each data point. This approach allows the data, which may initially appear as discrete points, to be interpreted as a coherent structure, thereby enabling the extraction of geometric and topological characteristics.

TDA has been reported to be effective in understanding the complex spatial and morphological dynamics of infectious disease spread. For example, Taylor et al. [23] demonstrated that TDA can be used to capture spatial and shape-based features of contagion processes, potentially contributing to the prediction and control of disease outbreaks.

Belchi et al. demonstrated that TDA is effective in identifying characteristics in medical images that are imperceptible to the human eye, exemplified by their analysis of the geometric structure of airways in computed tomography scans of patients with chronic obstructive pulmonary disease [24]. Garside et al. applied TDA to retinal vascular images for extracting imaging features using the convex peeling method [25] followed by machine learning methods to differentiate patients with diabetic retinopathy from healthy controls [26]. TDA holds potential as a diagnostic tool for NPSLE by identifying features that could serve as imaging biomarkers from brain MRI images to distinguish between patients with NPSLE and non-NPSLE. Despite this potential, no studies have integrated brain imaging analyses using TDA with clinical data. Consequently, this study aimed to identify discriminative biomarkers for NPSLE by applying TDA to brain MRI scans and quantitatively evaluating the diagnostic utility of each imaging feature.

## Materials and methods

### Patient cohort and imaging protocol

This retrospective study included 31 patients with NPSLE who were admitted to Nagasaki University Hospital between January 1, 2005, and August 31, 2020. All patients met at least four of the 11 revised criteria for SLE classification as defined by the American College of Rheumatology [27]. The neuropsychiatric symptoms were systematically evaluated based on the American College of Rheumatology nomenclature and case definitions for NPSLE (1999), incorporating neurological examinations, laboratory tests, and neuroimaging, including brain MRI. Subsequently, attribution of these symptoms to NPSLE was determined by consensus among multiple rheumatologists and psychiatrists, in accordance with the Italian Attribution Algorithm [28].

Exclusion criteria encompassed the absence of brain MRI data, as MRI-based analysis was essential, and a prior history of neuropsychiatric symptoms in patients with SLE to avoid overlap between NPSLE and non-NPSLE groups.

For comparative analysis, fluid-attenuated inversion recovery (FLAIR) brain MRI scans from 30 patients with NPSLE (NPSLE group) and 30 patients with SLE without neuropsychiatric symptoms (non-NPSLE group) were evaluated. MRI scans were obtained during the initial visit to Nagasaki University Hospital, or the initial diagnosis of NPSLE or SLE evaluation. The details of the MRI equipment used are provided in S1 Table.

### Data collection

We retrospectively reviewed medical records at the time of brain MRI to gather detailed demographic, clinical, and laboratory data. Demographic variables included age, sex, comorbidity history, and current prednisolone dosage. Clinical data included white blood cell count, hemoglobin, platelet count, blood urea nitrogen, creatinine, total protein, albumin, C-reactive protein, complement component C3 and complement component C4, 50% hemolytic unit of complement (CH50), antinuclear antibody titer, anti-Smith antibody titer, anti-double-stranded DNA antibody titer, anti-cardiolipin antibody level, anti-β2 glycoprotein 1 complex antibody level, and lupus anticoagulant status. The period during which data was accessed was from 22nd Dec 2020–5th Jun 2021.

### Topological data analysis

TDA encompasses a suite of methods for identifying and quantifying topological features within datasets. A key method within TDA, persistent homology, tracks the persistence of data features across multiple scales. Persistent homology is known for its robustness to noise, independence from dimensionality and coordinate systems, and ability to succinctly represent qualitative features of input data [29]. This technique has been successfully applied to various imaging datasets. In this study, we applied the methodology of Garside et al. [25] based on the Henderson’s topological features [26] to brain MRI scans and assessed their potential as imaging biomarkers to differentiate patients with NPSLE from those with non-NPSLE.

Persistent homology captures the evolution of topological structures as a function of varying threshold values in a two-dimensional lattice derived from each MRI scan. These features are categorized as 0-dimensional (connected components) and 1-dimensional (holes) [25]. Let z(x) denote the value of a random field at a location x on a two-dimensional lattice. For any real threshold t, the corresponding lower-leve$F_{t}=\{x:\;z(x)\leq t\},$l set is the collection of locations whose field values do not exceed t. As t increases from the minimum field value, these nested sets create a filtration that persistent homology uses to track how the field’s topology changes by varying the threshold value. Although persistent homology is usually developed in terms of simplicial complexes, in the present context we only need to monitor two kinds of topological features: connected components and holes. The connected component is a group of one or more pixels in the lower level set that are connected to each other. The hole is a group of one or more pixels that are not in the level set, are connected to each other but are isolated from other pixels that are also outside the level set. As the threshold value t increases, components and holes are born and die. For fields in two dimensions, a new component is born at a local minimum of the field z(x) and a hole dies at a local maximum. The persistence diagram is a scatterplot of birth levels against death levels for the components or the holes. See [25] and [26] for further details and illustrations.

Feature engineering is conducted to transform the persistence diagram into features that can be directly applied to machine learning models. However, the resulting features are typically high-dimensional [30] and difficult to handle, especially when the sample size is small. Henderson et al. [26] proposed a feature engineering method to summarize the persistence diagram into a small number of features that are easy to interpret and compare. They proposed using the 100α% convex peel to extract the general shape of the persistence diagram by peeling successive convex hulls until only a pre-scribed proportion (α) of points remain without undue influence of either outliers or the mass of points near the boundary.

**Fig 1:**
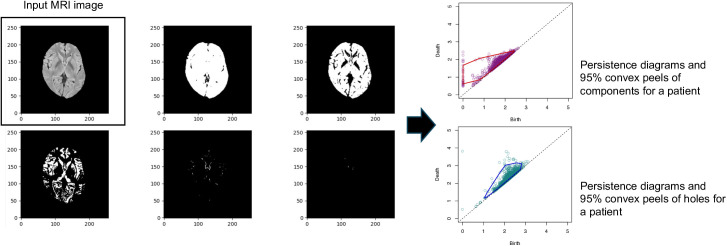
Persistence diagrams generated using Dionysus 2 software, alongside a scatter plot showing the birth and death times of topological components (connected components and holes) as a function of color variation in patient brain MRI scans. MRI: magnetic resonance imaging.

Although Garside et al. [25] applied machine learning methods to the Henderson’s features generated from 99%, 95%, and 90% convex peels, we only considered the intermediate convex peel of 95% by visual inspection to capture the shape of persistence diagrams in order to reduce redundancy between features because our focus is on identifying potential biomarkers that can be used in a simpler logistic regression model. Specifically, we considered the following seven topological features produced from each persistence diagram: 1 and 2. the two centroid coordinates (centroidx and centroidy): 3. the perimeter; 4. the area; 5. the filamentarity, which is defined as which measures how long and thin the convex peel is. In addition, we included: 6. the number of points of the persistence diagram (npoints) and 7. the average lifetime (lifetime), i.e., death time − birth time. Similar to Garside et al. [25], we applied the above Henderson’s topological features to discriminate brain images between patients with NPSLE and those with non-NPSLE. The procedure for extracting imaging features from brain MRI data involved the following steps: 1 and 2. the two centroid coordinates (centroidx and centroidy): 3. the perimeter; 4. the area; 5. the filamentarity, which is defined as which measures how long and thin the convex peel is. In addition, we included: 6. the number of points of the persistence diagram (npoints) and 7. the average lifetime (lifetime), i.e., death time − birth time. Similar to Garside et al. [25], we applied the Henderson et al. [26] ‘s topological features to discriminate brain images between patients with NPSLE and those with non-NPSLE. The procedure for extracting imaging features from brain MRI data involved the following steps: 1) Extracting the central slice from a collection of all slices of 3D brain MRI images for each patient (slices the brain horizontally from the top of the brain to the bottom); 2) Converting the central slice to grayscale with a resolution of 256 × 256 pixels; 3) Applying skull stripping using a deep learning-based algorithm developed by Buda et al. [31]; 4) Standardizing the grayscale values within the brain region by subtracting the mean and dividing by the standard deviation, with minimum values set to zero and zero values assigned to regions outside the brain; 5) Generating persistence diagram for each image using the lower star filtration method within the Dionysus 2 software package https://www.mrzv.org/software/dionysus2/tutorial/lower-star.html, capturing both 0-dimensional and 1-dimensional features [25]; 6) Obtaining 95% convex peels of the persistence diagrams for the both 0-dimensional and 1-dimensional features using the “plothulls” function in R package “aplpack”. Exemplified processing steps from an image of the brain MRI scan are depicted in Fig 1. The above procedures were independently applied to each patient’s brain MRI scan, resulting in two persistence diagrams per scan.

Subsequently, the seven summary statistics or features derived from the 95% convex peels of these diagrams were calculated. We evaluated these imaging features for their potential to discriminate between patients with NPSLE and non-NPSLE.

### Statistical analysis

Categorical variables were expressed as counts and percentages, while continuous variables were presented as medians with interquartile ranges (IQR) due to non-normal distribution. The Wilcoxon rank-sum test was employed for group comparisons of continuous variables. Logistic regression analysis was performed using the Wald test to assess associations between variables and NPSLE status, with adjustments for potential confounders. All statistical analyses were conducted using JMP (Student edition 18.2.0, Pro16, 17; SAS Institute, Cary, NC, USA), the statistical package R (version 4.5.0; available as free download from https://www.r-project.org/) and GraphPad Prism 9 (GraphPad Software, San Diego, CA, USA). Statistical significance was set at p-value < 0.05.

### Ethics

All researchers involved in this study conducted the research in accordance with the Declaration of Helsinki (revised in October 2013) and the “Ethical Guidelines for Medical and Health Research Involving Human Subjects”, issued by the Ministry of Education, Culture, Sports, Science and Technology and the Ministry of Health, Labour and Welfare (partially amended on February 28, 2017). The study was approved by the Clinical Research Ethics Committee of Nagasaki University Hospital (Approval Reference: 20122133).

Informed consent was obtained from all participants. Our study used samples and information obtained through routine medical practices and did not involve any interventions or invasive procedures. Given the possibility that some participants had already discontinued visits or were unable to fully understand the study due to neuropsychiatric symptoms, we ensured appropriate opportunities as follows. We publicly disclosed the study outline on the website of the Clinical Research Center of Nagasaki University Hospital. This procedure was in accordance with the Ethical Guidelines for Medical and Health Research Involving Human Subjects. The research participants had the opportunity to opt out of the use of their samples and information.

For handling samples and data, a unique identification code unrelated to personal information was assigned to each participant. In addition, all patient identifiers, including names and dates of birth, were removed from DICOM headers. A correspondence table linking the identification codes to personal identifiers was created and securely stored in accordance with the ethics protocol approved by the ethics committee.

## Results

### Patient characteristics

We analyzed the demographic and clinical characteristics of 30 patients with NPSLE and 30 with non-NPSLE. The median age (IQR) at the time of brain MRI was 39.5 (28.5–47.3) years in the NPSLE group and 44.5 (30.8–54.3) years in the non-NPSLE group (**p* *= 0.18). The NPSLE group comprised 22 (73%) females, while the non-NPSLE group had 25 (83%) females (**p* *= 0.35). The median disease duration (IQR) was 3.5 (0.75–11) in NPSLE group and 0 (0–7) in non-NPSLE group (*p* = 0.011). The median prednisolone for internal use (IQR) was 15 mg/day (5.94–20) in NPSLE group and 9 mg/day (0–30) in non-NPSLE group. A history of cerebrovascular disease was present in 9 (30%) patients in the NPSLE group and in 3 (10%) in the non-NPSLE group. Median levels (IQR) of serum anti-ds-DNA antibodies (U/mL), serum anti-Sm antibodies (U/mL), serum complement component C3 (mg/dL) and serum complement C4 (mg/dL), and CH50 (mg/dL) were 9.45 (2.6–49.9), 2.85 (0.68–23.5), 69.6 (54.8–88.9), 13.3 (9.3–20.6), and 30.6 (24.3–37.7), respectively, in the NPSLE group, compared to 15.6 (5.33–40.5), 6.9 (1.95–86.4), 60.4 (44.1–74.6), 9.1 (6.3–11.8), and 24.4 (19.8–31.9), respectively, in the non-NPSLE group. There were no significant differences in autoantibodies associated with antiphospholipid antibody syndrome between the NPSLE and non-NPSLE groups (Table 1). In NPSLE group, 17 (57%) patients had headache, and seizure, cerebrovascular disease, acute confusional state, anxiety disorder, mood disorder, cognitive dysfunction, psychosis, autonomic disorder, neuropathy and myathenia were present in 5 (17%), 9 (30%), 3 (10%), 6 (20%), 10 (33%), 7 (23%), 6 (20%), 4 (13%), 3 (10%) and 5 (17%) patients, respectively (Table 2).

**Table 1:** Demographic and clinical characteristics of patients with and without neuropsychiatric systemic lupus erythematosus (NPSLE).

	NPSLE (n = 30)	non-NPSLE (n = 30)	*p*-value
Age, yr, median (IQR)	39.5 (28.5–47.3)	44.5 (30.8–54.3)	0.18
Female, n, (%)	22 (73%)	25 (83%)	0.35
Disease duration, yr, median (IQR)	3.5 (0.75–11)	0 (0–7)	0.011
Prednisolone for internal use, mg/day, median (IQR)	15 (5.94–20)	9 (0–30)	0.18
Complication, n, (%)			
Cerebrovascular disease, n, (%)	9 (30%)	3 (10%)	0.053
Diabetes mellitus, n, (%)	4 (13%)	6 (20%)	0.49
Hypertension, n, (%)	12 (40%)	11 (37%)	0.79
Myocardial infarction, n, (%)	0 (0%)	1 (3%)	0.31
Nephropathy, n, (%)	9 (30%)	14 (47%)	0.18
Laboratory data, median (IQR)			
White blood cell, μL	5550 (3975–7375)	4700 (3650–5800)	0.13
Hemoglobin, g/dL	11.7 (10.1–12.6)	11.6 (9.93–12.7)	0.99
Platelet, μL	19.3 (12.7–25.4)	19.2 (10.4–22.6)	0.55
BUN, mg/dL	14.5 (10.8–19.0)	11.5 (9.75–20.3)	0.30
Creatinine, mg/dL	0.72 (0.58–0.97)	0.71 (0.54–0.85)	0.54
Total protein, g/dL	6.65 (6.18–7.43)	7.0 (6.3–7.83)	0.37
Albumin, g/dL	3.7 (3.1–4.1)	3.6 (3.0–4.0)	0.49
CRP, mg/dL	0.09 (0.04–0.44)	0.27 (0.04–1.32)	0.38
Antinuclear antibody-positive, n, (%)	18 (86%)	28 (97%)	0.16
Anti-ds-DNA antibody, IU/mL	9.45 (2.6–49.9)	15.6 (5.33–40.5)	0.18
Anti-Sm antibody, U/mL	2.85 (0.68–23.5)	6.9 (1.95–86.4)	0.045
Anti-cardiolipin antibody, U/mL	5.6 (1.48–10.1)	7.0 (1.4–18.3)	0.62
Anti-cardiolipin β glycoprotein 1 complex antibody, U/mL	1.2 (1.2–1.95)	1.2 (1.2–2.18)	0.90
Lupus anticoagulant	1.16 (1.06–1.36)	1.16 (1.03–1.4)	0.88
C3, mg/dL	69.6 (54.8–88.9)	60.4 (44.1–74.6)	0.039
C4, mg/dL	13.3 (9.3–20.6)	9.1 (6.3–11.8)	0.012
CH50, mg/dL	30.6 (24.3–37.7)	24.4 (19.8–31.9)	0.035

BUN, blood urea nitrogen; CRP, C-reactive protein; C3, complement component C3; C4, complement component C4; CH50, 50% hemolytic unit of complement; anti-ds-DNA, anti-double-stranded DNA; IQR, interquartile range; NPSLE, neuropsychiatric systemic lupus erythematosus; SLE, systemic lupus erythematosus

**Table 2:** The specific neuropsychiatric symptoms in NPSLE group.

NPSLE symptoms, n, (%)	NPSLE (n = 30)
Headache, n, (%)	17 (57%)
Seizure, n, (%)	5 (17%)
Celebrovascular disease	9 (30%)
Acute confusional state, n, (%)	3 (10%)
Anxiety disorder, n, (%)	6 (20%)
Mood disorder, n, (%)	10 (33%)
Cognitive dysfunction, n, (%)	7 (23%)
Psychosis, n, (%)	6 (20%)
Autonomic disorder, n, (%)	4 (13%)
Neuropathy, n, (%)	3 (10%)
Myasthenia, n, (%)	5 (17%)

### Comparison of imaging features between NPSLE and non-NPSLE groups

Using the TDA procedure previously described, we generated persistence diagrams and associated summary statistics (imaging features) for each patient in the NPSLE and non-NPSLE groups. We assessed these imaging features by comparing their distributions between the NPSLE and non-NPSLE datasets. While no significant difference was observed in the perimeter of the components (median [IQR]: 7.53 [6.91–8.19] vs. 7.15 [6.73–7.80], **p* *= 0.081), a significant difference was found in the perimeter of the holes (7.21 [5.62–8.60] vs. 6.43 [4.92–7.70], **p* *= 0.046), with longer median perimeter values in the NPSLE group compared to the non-NPSLE group. Additionally, a significant difference was observed in the area of the holes (1.32 [0.99–1.86] vs. 1.07 [0.75–1.39], **p* *= 0.032), with larger median area values in the NPSLE group than in the non-NPSLE groups (S2 Table). Additionally, we calculated the Cohen’s d for each imaging feature. The values for the perimeter and area of the holes were above 0.5 (S2 Table). We conducted a post-hoc power analysis and confirmed that the statistical powers of the perimeter and area of the holes were 0.54 and 0.56, respectively, both of which were below the commonly accepted threshold of 0.8.

### Clinical parameters discriminating between NPSLE and non-NPSLE groups

Complement titers (C3, C4 and CH50) were lower in the non-NPSLE group than in the NPSLE group, indicating that the non-NPSLE group had higher SLE disease activity (Table 1). Among the NPSLE group, 4 patients exhibited only diffuse manifestations of central nervous system symptoms (diffuse NPSLE group), while 11 patients showed only focal manifestations (focal NPSLE group). A comparison of serum C3, C4, and CH50 levels between the diffuse and focal groups showed no significant differences (**p* *= 0.43 for C3, **p* *= 0.60 for C4 and **p* *= 0.90 for CH50; S1 Fig). However, the median values of C3 and CH50 were lower in the diffuse NPSLE group (S1 Fig. left and right).

**Fig 2:**
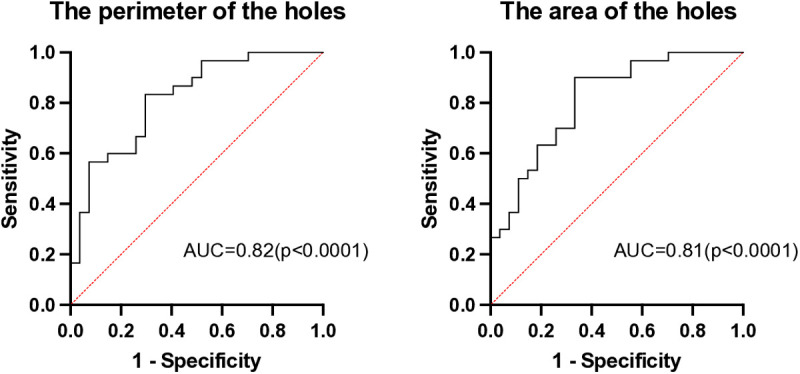
Receiver operating characteristic (ROC) curve from multiple logistic regression analysis combining the perimeter and area of the holes, age, history of cerebrovascular disease, and 50% hemolytic unit of complement (CH50) levels.

Multiple logistic regression analyses, which included age, history of cerebrovascular disease, and CH50 alongside the perimeter and area of the holes, revealed a significant association with NPSLE (odds ratio [OR]: 1.67, 95% confidence interval [CI]: 1.07–2.63, **p* *= 0.025 for perimeter, OR: 4.42, 95% CI: 1.35–19.6, *p* = 0.026 for area; Tables 3 and 4). To assess the robustness of the odds ratios, we applied the bootstrap method (10,000 resamples, percentile method) to re-estimate the 95% confidence intervals. The bootstrap CI for perimeter was 1.17–4.42 and for area, 1.41–97.2 (Table 5). Both the perimeter and area of the holes demonstrated a high area under the curve (AUC), exceeding 0.8 (Fig 2).

**Table 3:** Multiple logistic regression analysis incorporating the perimeter of the holes, history of cerebrovascular disease, age, and 50% hemolytic unit of complement (CH50) levels.

	Odds ratio	95% CI	p-value	BH-adjusted p-value
Perimeter1	1.67	1.07–2.63	0.025	0.035
Cerebrovascular disease	8.46	1.24–57.5	0.029	0.035
Age	0.93	0.88–0.99	0.014	0.035
CH50	1.07	1.00–1.14	0.035	0.035

CH50, 50% hemolytic unit of complement; CI, confidence interval; perimeter1, the arc length of 95% convex peels of the holes; BH, The Benjamini-Hochberg method

**Table 4:** Multiple logistic regression analysis incorporating the area of the holes, history of cerebrovascular disease, age, and 50% hemolytic unit of complement (CH50) levels.

	Odds ratio	95% CI	p-value	BH-adjusted p-value
Area1	4.42	1.35–19.6	0.026	0.035
Cerebrovascular disease	9.74	1.61–93.0	0.024	0.035
Age	0.93	0.87–0.98	0.021	0.035
CH50	1.07	1.01–1.15	0.036	0.036

CH50, 50% hemolytic unit of complement; CI, confidence interval; area1, the area of 95% convex peels of the holes; BH, The Benjamini-Hochberg method

**Table 5:** Bootstrap estimates of odds ratios and 95% confidence intervals for the perimeter and area of the holes.

	Odds ratio (Bootstrap)	95% CI (Bootstrap, percentile method)
Perimeter1	1.76	1.17-4.42
Area1	4.31	1.41-97.2

CI, confidence interval; perimeter1, the arc length of 95% convex peels; area1, the area of 95% convex peels of the holes

We also added the treatment status and disease duration in the logistic regression analysis as covariates. Both the perimeter and area of the holes remained significant (odds ratio [OR]: 1.70, 95% confidence interval [CI]: 1.03–2.80, **p* *= 0.037 for perimeter, OR: 4.59, 95% CI: 1.13–18.6, *p* = 0.033 for area; S3 and S4 Tables)

Multiple logistic regression analyses, which included age and CH50 alongside the perimeter and area of the holes, revealed a significant association with NPSLE (odds ratio [OR]: 1.77, 95% confidence interval [CI]: 1.20–2.90, **p* *= 0.0099 for perimeter, OR: 4.91, 95% CI: 1.60–20.0, *p* = 0.012 for area; S5 and S6 Tables).

A comparison of the perimeter and area of the holes between the diffuse and focal NPSLE groups revealed that both values were greater in the diffuse NPSLE group (**p* *= 0.050 for perimeter, **p* *= 0.019 for area; S2 Fig.)

### Assessing the impact of MRI equipment variability

**Fig 3:**
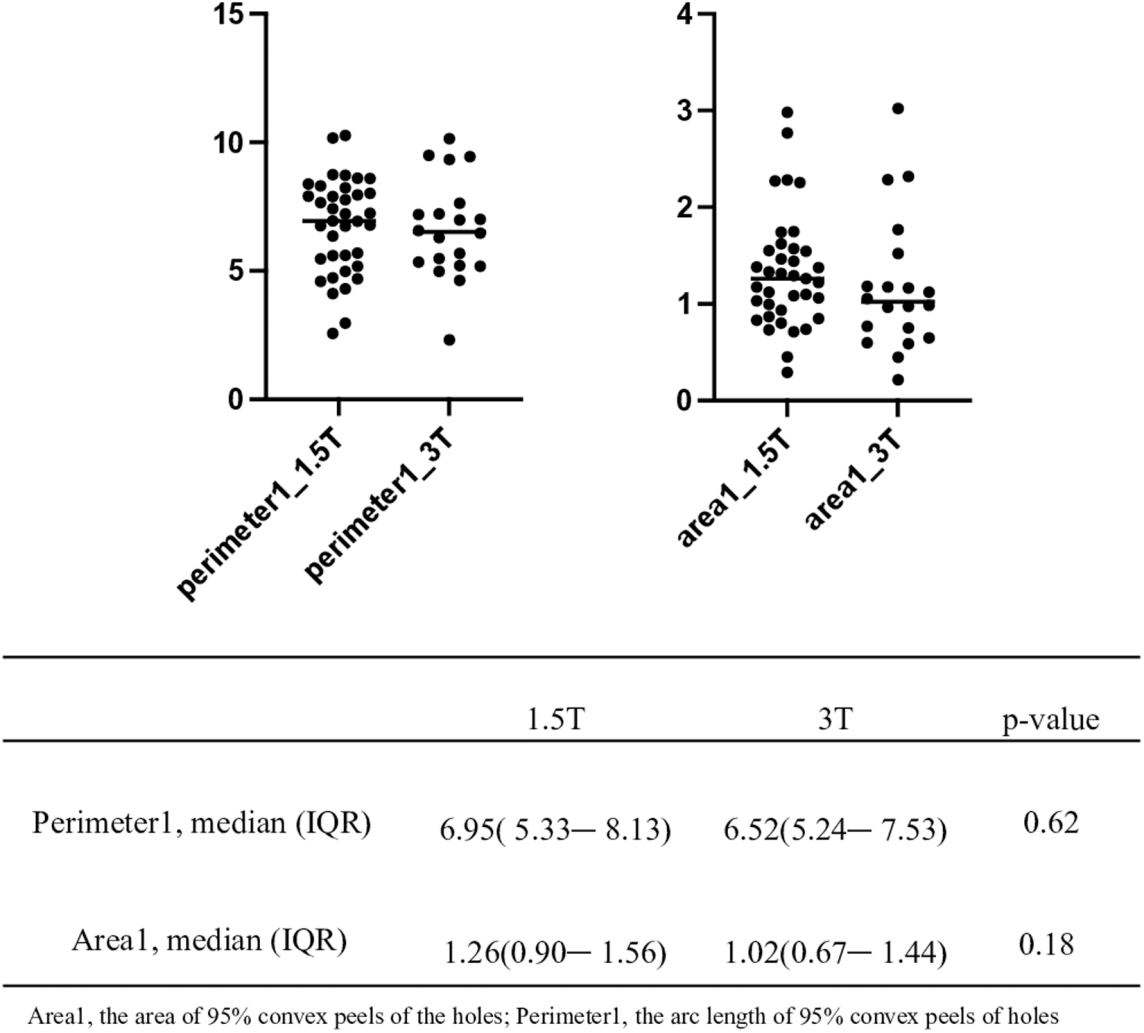
The left figure shows boxplots comparing the perimeter of the holes measurements between the 1.5T MRI images and the 3T MRI images. The right figure shows boxplots comparing the area of the holes measurements between the 1.5T MRI images and the 3T MRI images. The *p*-values are from the Wilcoxon rank sum test.

**Fig 4:**
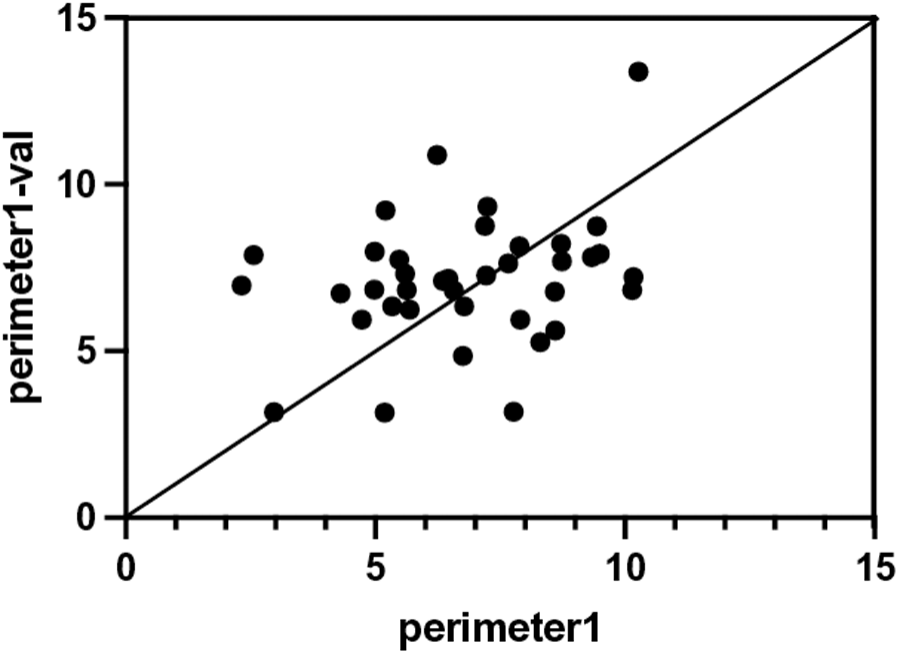
Scatter plot comparing the perimeter of the holes measurements between the analysis images and validation images, with all data points distributed around the diagonal line, indicating a close agreement.

Given the findings above, it remains unclear whether the perimeter and area of the holes metrics would remain consistent across different MRI machines. To address this, we compared the perimeter and area of the holes between the 1.5T MRI image group and the 3T MRI image group. No significant differences were found (**p* *= 0.62 for perimeter, **p* *= 0.18 for area; Fig 3). Additionally, we tested whether variations in MRI equipment would affect our results by capturing images of the same patients using different MRI machines. Similar results were observed (Fig 4). However, because many patients in the SLE group underwent brain MRI scanning only once, validation was limited to 16 patients in the SLE group and 22 patients in the NPSLE group, introducing potential bias. Therefore, while our results suggest that similar findings could be obtained using different MRI equipment, this conclusion remains tentative.

### A Comparison of imaging features between NPSLE patients with and without WMHs on brain MRI

In the NPSLE group, WMHs were observed on brain MRI in 20 patients (WMHs group), while no WMHs were observed in 10 patients (non-WMHs group). A comparison of the perimeter and area of the holes between the WMHs group and the non-WMHs group showed no significant differences (**p* *= 0.11 for perimeter, **p* *= 0.62 for area; S3 Fig).

## Discussion

In our explanatory analyses, we found that the perimeter and area of the 95% convex peel of the holes were significantly longer and larger, respectively, in the NPSLE group compared to the non-NPSLE group. This suggests that these topological features could serve as reliable imaging biomarkers for distinguishing patients with NPSLE from non-NPSLE using TDA.

However, the statistical power of the perimeter and area of the holes were 0.54 and 0.56 by a post-hoc power analysis. We acknowledge that the current study is underpowered and therefore position this work as an exploratory study.

Nonetheless, moderate effect sizes were observed for the identified imaging features: the perimeter of the holes (p = 0.046, Cohen’s d = 0.55) and the area of the holes (p = 0.032, Cohen’s d = 0.57). This study represents a novel attempt to extract quantifiable imaging features from brain MRI scans of patients with NPSLE—a rare condition—using TDA, a data science method capable of identifying complex spatial patterns that may not be visually apparent to human observers. We recognize that validating the potential of these novel imaging features as NPSLE biomarkers is critically important, and this is currently being addressed in an ongoing multicenter study, which currently involves eight participating institutions.

Next, we discuss the selection of clinical variables for the regression models. One of the pathogeneses of NPSLE was thought to be related to the destruction of the blood-brain barrier due to vascular damage [32] and cerebrovascular disease is a symptom of NPSLE in the ACR diagnostic criteria. This is why we selected history of cerebrovascular disease for the regression model. The involvement of the complement system in NPSLE pathogenesis has been well-documented in previous studies [33,34]. Ota et al. [32] reported that the activation of immune complexes and complement is related to the damage of vascular endothelial cells in the ischemic process in NPSLE. Therefore, we selected CH50 as the independent variable. SLE frequently occurs in young women of childbearing age, and NPSLE often develops within a short period of time after the onset of SLE. Furthermore, atrophy and WMHs are known to be affected by aging. Based on these, we included age for the regression models. Even after adjusting for age, history of cerebrovascular disease, and CH50 levels, the perimeter and area of the holes remained significantly associated with NPSLE. The high AUC values (above 0.8) further underscore their potential as valuable biomarkers.

We applied the Benjamini-Hochberg procedure to adjust for the false discovery rate across all covariates used in the multivariable logistic regression models, including the perimeter and area of the holes, age, 50% hemolytic unit of complement levels, and history of cerebrovascular disease. The adjusted p-values for all variables remained below the 0.05 threshold, indicating that their statistical significance was maintained after correction. However, the bootstrap CI for the perimeter of the holes was 1.17–4.42 and for the area of the holes was 1.41–97.2 (Table 5). Although these intervals are relatively wide, the lower bounds remained above 1, supporting a potential association with NPSLE. However, the wide range reflects uncertainty in the estimates, and the findings should be interpreted with caution.

We believe that WMHs were partly included in our analysis. Previous research has shown that the size and distribution of WMHs differ between patients with NPSLE and SLE [35]. For instance, NPSLE patients with central nervous system symptoms often present with significantly more lesions above 10 mm on brain MRI compared to those without neuropsychiatric symptoms [12]. Additionally, a marked reduction in prefrontal white matter integrity has been observed in patients with NPSLE relative to those without NPSLE [36]. WMHs are also associated with reduced cerebral blood flow [37]. Both patients with NPSLE and non-NPSLE exhibit decreased cerebral blood flow compared to healthy controls [38]. Notably, a significant reduction in cerebral blood flow has been documented in the white matter of patients with NPSLE who have unremarkable brain MRI findings [39]. Furthermore, distinct brain perfusion patterns have been observed in patients with NPSLE and non-NPSLE [40]. These findings are consistent with our observation that a higher proportion of patients with NPSLE had a history of cerebrovascular disease compared to patients with non-NPSLE.

The imaging features obtained by TDA, the perimeter and area of the holes, are derived from the 95% convex peel of the persistence diagram, which plots the birth and death times of topological holes created by merging data structures such as WMHs after grayscale transformation of the brain MRI images. In the NPSLE group, there was a tendency for the perimeter and area of the holes to be larger than in the non-NPSLE group. According to Henderson et al. [26], when a dataset contains lots of short-lived features (noise), the 95% convex peel tends to be more linear in shape. In contrast, a larger number of long-lasting features results in a more circular 95% convex peel. Analogously, we would expect that the presence of more long-lasting features may result in a larger area and similarly a longer perimeter. Thus, the observation that NPSLE patients exhibit such a large area and a longer perimeter may suggest the presence of more long-lasting features, implying larger WMHs that are not attributable to noise.

Furthermore, the death of a hole is associated with a local maximum according to Henderson et al. [26] Given this, a greater perimeter and area of the holes observed in NPSLE patients would imply frequent birth and death of topological holes across different pixel value thresholds, which in turn implies the presence of many WMHs in NPSLE. Ingres et al. reported that WMHs in NPSLE patients are larger in volume and exhibit more complex shapes compared to those in non-NPSLE patients [14]. The present findings are consistent with these observations.

Box-and-whisker plots and medians in S3 Fig. show that the distribution of TDA features for the WMHs and non-WMHs groups tended to be consistently larger for the WMHs group. The difference was not significant (p = 0.11 for perimeter, p = 0.062 for area), possibly due to the small sample size.

Cerebrovascular disease and related risk factors such as hypertension and diabetes may potentially confound the interpretation of topological features such as perimeter, area, and hole count, as these comorbidities could influence white matter hyperintensity, which in turn contributes to these topological metrics. To account for the confounding, we performed multivariate logistic regression analysis adjusting for the history of cerebrovascular disease, age, and complement levels, and still observed a significant association of area and perimeter of the holes with NPSLE (Tables 3–4).

Multiple logistic regression analyses, which included age and CH50 alongside the perimeter and area of the holes, revealed a significant association with NPSLE (S5 and S6 Tables). Furthermore, to quantitatively evaluate the impact of cerebrovascular disease on the TDA-derived features—perimeter and area—we conducted additional multiple logistic regression analyses that excluded cerebrovascular disease. The changes in log-odds for the perimeter and area of the holes estimated from the logistic regression were 6.6% and 10.2%, respectively, suggesting that confounding has a minor impact on their interpretation.

Studies in recent years have proposed that complement activation is involved in the pathophysiological processes of central nervous system inflammation and neurodegenerative diseases and is considered one of the factors involved in the pathogenesis of NPSLE [41]. Magro-Checa et al. reported the following: C3 and CH50 were significantly lower in NPSLE patients compared to SLE patients without NPSLE, and especially in diffuse NPSLE. No differences were found in focal NPSLE. An association between NPSLE patients and lower values of C4 was not found. However, low C4 levels were more prevalent in diffuse NPSLE. No association was found with focal NPSLE [34].

In our study, the Complement titers (C3, C4 and CH50) were lower in the non-NPSLE group than in the NPSLE group, indicating that the non-NPSLE group had higher SLE disease activity (Table 1). This finding may reflect the predominance of cases with only focal manifestations in the NPSLE group. A comparison of C3, C4 and CH50 levels between the diffuse and the focal NPSLE showed no significant differences. However, the median C3 and CH50 levels were higher in the focal NPSLE group than in the diffuse NPSLE group. Although the result is not statistically significant due to the small sample size, we believe this result shows a trend consistent with the findings of Magro-Checa et al. [34]

Ingres et al. reported that WMHs in diffuse NPSLE were larger and morphologically more complex [14]. This may correspond with our result showing that both the perimeter and area of the holes were greater in diffuse than in focal NPSLE. Further investigation is needed to elucidate the relationship between TDA-based imaging features and the underlying pathological mechanisms of NPSLE.

Furthermore, we believe that the TDA-derived biomarkers may be potentially useful for predicting the development of NPSLE in SLE patients. Silvagni et al. reported that diffusivity on brain MRI significantly increased in newly diagnosed SLE patients, suggesting that early white matter changes may occur even in the absence of neuropsychiatric symptoms [42]. Therefore, we believe that the analysis of brain MRI using TDA might capture early changes in white matter structure and has a potential to predict the development of NPSLE in patients.

Kitagori et al. reported that cerebrospinal fluid levels of osteopontin—an immunomodulatory molecule related to inflammatory cell migration—were significantly elevated in NPSLE patients compared to non-NPSLE patients before treatment and decreased following therapy [43]. These findings suggest that immunological activity in NPSLE is modifiable by treatment, and may also imply that corresponding changes could be observed in brain MRI findings. Therefore, we believe that applying TDA to serial MRI scans may help detect subtle structural changes and offer a novel imaging biomarker for assessing treatment response and disease mechanisms.

Further research is needed to determine whether TDA analysis has these possibilities.

From a methodological perspective, TDA offers several advantages. First, TDA is an unsupervised machine-learning technique that does not require ground-truth labels, making it particularly suitable for diseases like NPSLE, where diagnostic criteria are not fully developed. Second, as previously mentioned, TDA is robust against perturbations in input data and is independent of dimensions and coordinates. Brain MRI scans may vary in quality depending on the scanner used, including differences in the number of slices and resolution. These variations can complicate unified analysis; however, TDA’s robustness allows for the automatic capture of lesions, such as WMHs, from image data without the need for meticulous manual alignment of brain regions across patients. Therefore, TDA holds promise for the automated extraction of imaging biomarkers from brain MRI scans obtained using different MRI equipment, reducing the labor-intensive nature of manual processing. Our validation study also suggested a similarity of the imaging features obtained from different MRI equipment, but the small sample size limits the strength of conclusion. Regarding the methodology in making topological features, although we employed feature engineering through the convex peeling method to measure persistence diagrams recently proposed by Henderson et al. [26], this method may not always be reasonable in general. For instance, the convex peeling method removes points far away from the diagonal line if their proportion is small, even if such points show important characteristics of the persistence diagram. There is growing interest in identifying which summary statistics are most effective for experimental comparisons [44]. Improved summary of persistence diagrams could enhance the utility of TDA-derived imaging biomarkers.

Radiomics methods can be considered as a competing technique to imaging analysis using TDA. These methods enable the quantitative analysis of standard medical images using automated or semi-automated software, providing information that supports clinical decision-making such as diagnosis and prognosis [45].

Traditional radiomics methods, including texture analysis, often rely on handcrafted features derived from segmented images. These features can vary substantially depending on segmentation variability and imaging parameters [45,46]. In contrast, TDA does not require prior segmentation, allowing for fully automated analysis. By analyzing the persistence of topological features across multiple scales, TDA has the potential to distinguish true structural signals from noise, as noise-induced features tend to disappear quickly [47]. In our study, TDA-derived features were consistent across different MRI scanners (Fig 4), supporting its noise robustness. Moreover, TDA is effective even with small sample sizes [47], making it suitable for rare diseases such as NPSLE.

Next, we discuss the effect of the magnetic field strength of the MRI on the analysis using TDA. Stankiewicz et al. reported that high signal lesions were detected in 3T FLAIR images that were not detected in 1.5T [48]. The difference in magnetic field strength is thought to affect the detection of WMHs. However, we found no significant differences in the perimeter and area of the holes in our comparisons between 1.5T and 3T MRI image groups (Fig 3). One possibility is that our TDA-based algorithm could capture the NPSLE characteristics of the entire brain MRI image uncapable by the different magnetic field strengths. For instance, it has been reported that NPSLE patients not only have a large number and size of WMHs, but also have specific spatial characteristics, such as a more complex WMH shape in NPSLE patients compared with non-NPSLE patients [14]. The effects of field strengths on our algorithm and features need be validated using independent datasets.

In our study, we assessed whether variability in MRI equipment would affect the analysis and found that images acquired from the same patient using different MRI scanners yielded consistent results (Fig 4). Additionally, our method demonstrated robustness across different field strengths (S1 Table), indicating that the analysis is not significantly influenced by MRI hardware.

Although the true prevalence of NPSLE remains uncertain, estimates suggest that between 12% and 90% of SLE patients may develop neuropsychiatric symptoms [49]. In a multiethnic American cohort, 80% of SLE patients showed neuropsychiatric involvement [50], while a Finnish study reported a prevalence of 91% [51]. These findings suggest that the occurrence of NPSLE may be consistently high across diverse populations.

Based on this evidence, we consider our proposed diagnostic tool to be broadly applicable across different countries and populations.

We recognize that this study has the following limitations: 1) This study only suggests a possible new biomarker due to its small sample size, lack of statistical power, and lack of generalizability and reproducibility. 2) A diagnosis of NPSLE is not definitive. There is a presence of inter-facility bias, including bias related to diagnosis, the MRI equipment used, and the patient.

First, it was indeed challenging to recruit a larger number of patients because NPSLE is a rare disease. This study was designed as exploratory research. We recognize that the results do not guarantee reproducibility or generalizability. Reproducibility, in this context, refers to obtaining similar results in an independent cohort, which has not yet been achieved. Although the AUC of ROC was above 0.8 in this study, we recognize that an external validation using an independent cohort is essential to confirm the robustness of the model. To address these issues, we are currently conducting a multicenter study. We believe that this approach will be essential to strengthen the interpretation and reliability of the results.

Second, this study, being a single-center retrospective design, is subject to inherent limitations such as potential patient selection bias. In this study, we analyzed brain MRI images acquired from the same patients using different MRI scanners with TDA and found a certain degree of similarity in the extracted features (Fig 4). However, while we have conducted analyses using 1.5T and 3T MRI images, data from 7T MRI scanners have not been evaluated, and thus our investigation into the effects of scanner differences remains insufficient. All patients in this study were diagnosed based on the ACR criteria and the Italian Attribution Algorithm, but variability in diagnostic practices across centers also requires further examination.

We are currently conducting a multicenter study in collaboration with eight institutions in Japan. This ongoing study will enable us to evaluate the reproducibility and generalizability of the imaging biomarkers for NPSLE identified in the present work.

## Conclusion

This retrospective study identified novel imaging features specific to patients with NPSLE that were absent in patients with SLE without neuropsychiatric symptoms. By employing TDA, we demonstrated that combining age, history of cerebrovascular disease, and CH50 titers with the perimeter and area of the holes yielded significant associations in multiple logistic regression analyses. These findings underscore the potential of TDA as an effective imaging diagnostic tool for distinguishing patients with NPSLE from those with non-NPSLE.

## Supporting information

S1 Fig The figures show boxplots comparing each serum compliment level between the diffuse NPSLE and the focal NPSLE. The left: C3 levels, The center: C4 levels, The right CH50 levels.
(TIF)

S2 Fig The left figure shows boxplots comparing the perimeter of the holes measurements between the diffuse and focal NPSLE patients. The right figure shows boxplots comparing the area of the holes measurements between the diffuse and focal NPSLE patients.
(TIF)

S3 Fig The left figure shows boxplots comparing the perimeter of the holes measurements between the NPSLE patients with and without WMHs on brain MRI. The right figure shows boxplots comparing the area of the holes measurements between the NPSLE patients with and without WMHs on brain MRI.
(TIF)

S1 Table The details of the MRI equipment used. (DOCX)

S2 Table Comparative analysis of imaging features between the NPSLE and non-NPSLE groups. (DOCX)

S3 Table Multiple logistic regression analysis incorporating the perimeter of the holes, history of cerebrovascular disease, age, 50% hemolytic unit of complement (CH50) levels, disease duration and prednisolone for internal use. (DOCX)

S4 Table Multiple logistic regression analysis incorporating the area of the holes, history of cerebrovascular disease, age, 50% hemolytic unit of complement (CH50) levels, disease duration and prednisolone for internal use. (DOCX)

S5 Table Multiple logistic regression analysis incorporating the perimeter of the holes, age, 50% hemolytic unit of complement (CH50) levels. (DOCX)

S6 Table Multiple logistic regression analysis incorporating the area of the holes, age, 50% hemolytic unit of complement (CH50) levels. (DOCX)

S1 File Data. (XLSX)
