# Supplementary figures and images for: Novel imaging diagnosis of neuropsychiatric systemic lupus erythematosus using topological data analysis: A retrospective study
Source: PLoS One. 2025 Aug 13;20(8):e0329859. doi: 10.1371/journal.pone.0329859 (PMC12349068; doi:10.1371/journal.pone.0329859)

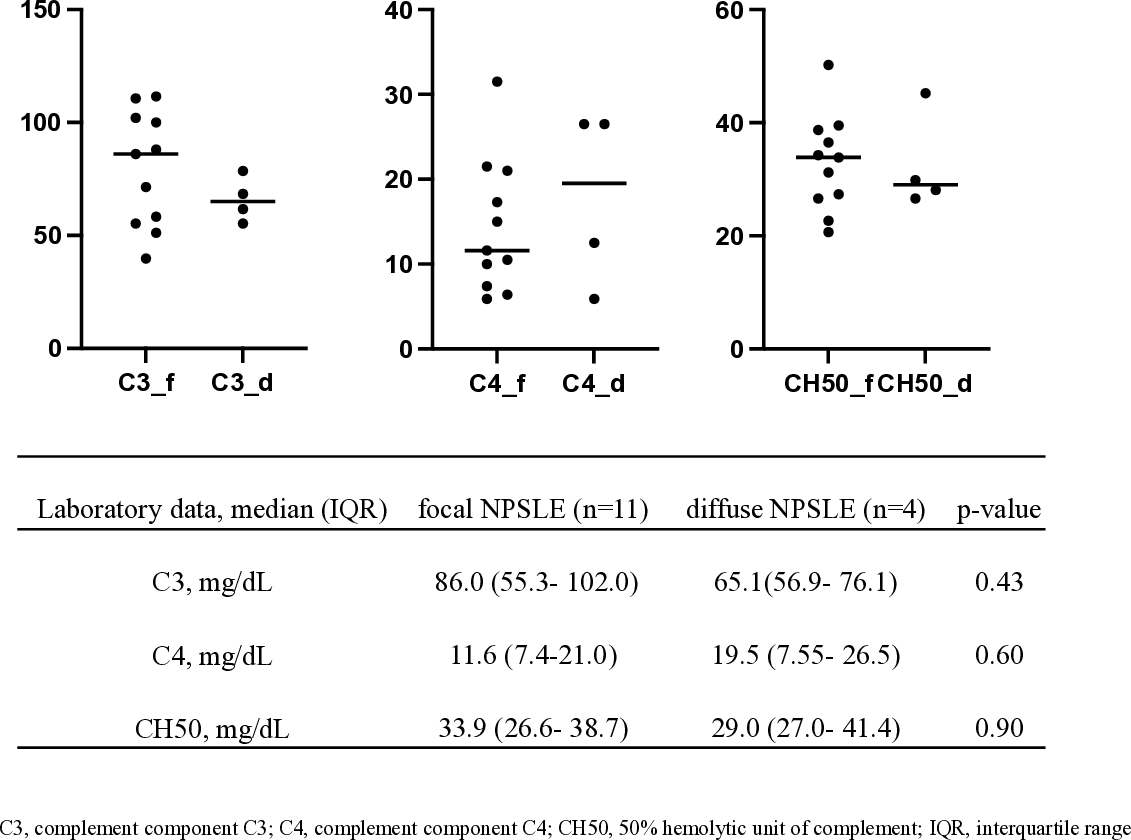

Supplement: S1 Fig — The left: C3 levels, The center: C4 levels, The right CH50 levels. (TIF) [file pone.0329859.s001.tif]

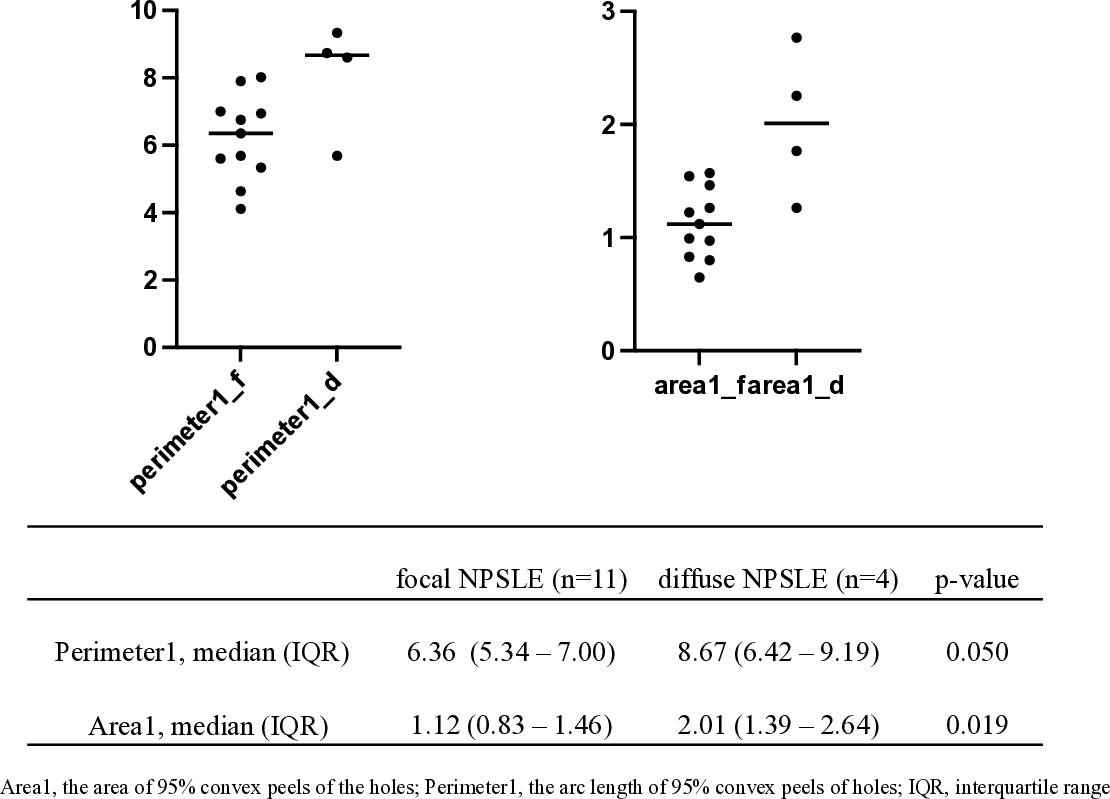

Supplement: S2 Fig — The right figure shows boxplots comparing the area of the holes measurements between the diffuse and focal NPSLE patients. (TIF) [file pone.0329859.s002.tif]

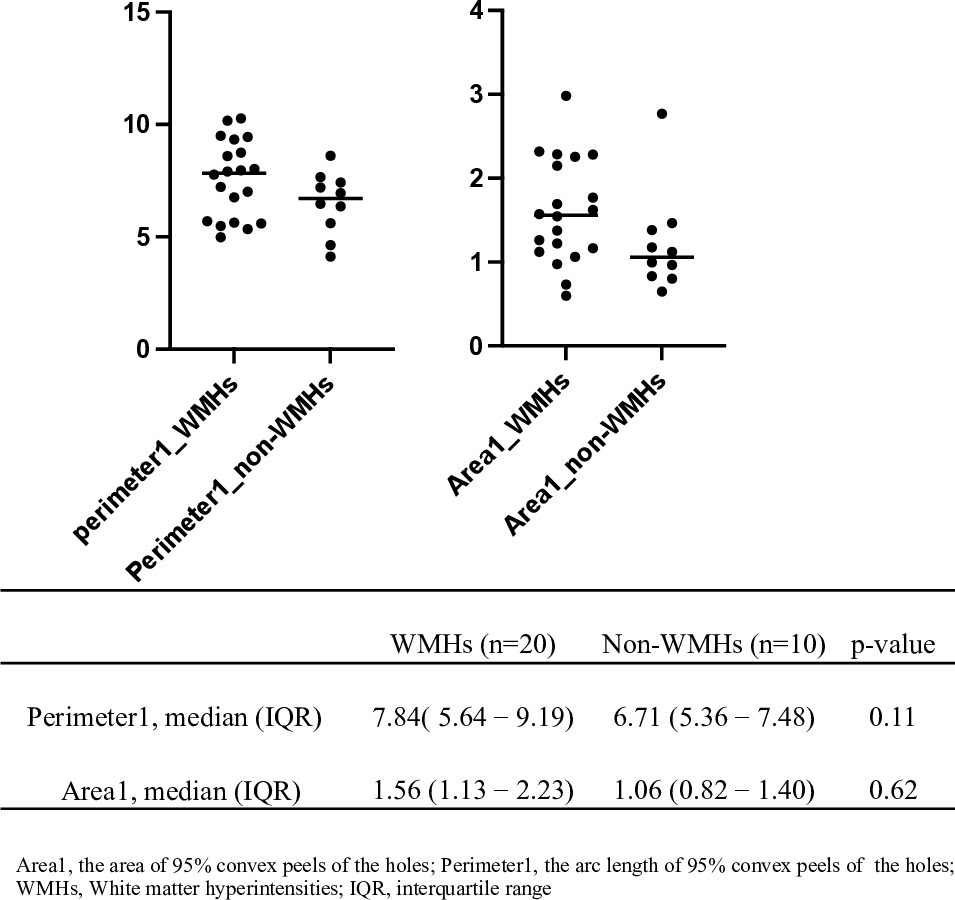

Supplement: S3 Fig — The right figure shows boxplots comparing the area of the holes measurements between the NPSLE patients with and without WMHs on brain MRI. (TIF) [file pone.0329859.s003.tif]
