# Supplementary material for: Novel imaging diagnosis of neuropsychiatric systemic lupus erythematosus using topological data analysis: A retrospective study
Source: PLoS One. 2025 Aug 13;20(8):e0329859. doi: 10.1371/journal.pone.0329859 (PMC12349068; doi:10.1371/journal.pone.0329859)
Supplement: S1 Table — (DOCX) [file pone.0329859.s004.docx]

**S1 Table. The details of the MRI equipment used**

| The name of the MRI equipment used | Field strength | The number of MRI images, n, (%)  (n=60) | The number of validation MRI images, n, (%)  (n=42) |
| --- | --- | --- | --- |
| Skyra, Siemens Healthcare GmbH, Erlangen, Germany | 3T | 5 (8%) | 3 (7%) |
| Avanto, Siemens Healthcare GmbH, Erlangen, Germany | 1.5T | 6 (10%) | 3 (7%) |
| SIGNA HDx, GE Healthcare, Waukesha, WI, US | 1.5T or 3T | 3 (5%) | 2 (5%) |
| SIGNA HDxt, GE Healthcare, Waukesha, WI, US | 1.5T or 3T | 26 (43%) | 22 (52%) |
| GENESIS_SIGNA, GE Healthcare, Waukesha, WI, US | 1.5T | 9 (15%) | 2 (5%) |
| Avanto_fit, Siemens Healthcare GmbH, Erlangen, Germany | 1.5T | 6 (10%) | 6 (14%) |
| MAGNETOM Vida, Siemens Healthcare GmbH, Erlangen, Germany | 3T | 1 (2%) | 1 (2%) |
| SIGNA Architect, GE Healthcare, Waukesha, WI, US | 3T | 1 (2%) | 3 (7%) |
| Unknown. | Unknown | 3 (5%) | 0 (0%) |
