# Supplementary material for: Novel imaging diagnosis of neuropsychiatric systemic lupus erythematosus using topological data analysis: A retrospective study
Source: PLoS One. 2025 Aug 13;20(8):e0329859. doi: 10.1371/journal.pone.0329859 (PMC12349068; doi:10.1371/journal.pone.0329859)
Supplement: S2 Table — (DOCX) [file pone.0329859.s005.docx]

**S2 Table.** **Comparative analysis of imaging features between the NPSLE and non-NPSLE groups**

|  | NPSLE (n=30) | Non-NPSLE (n=30) | p-value | Cohen’s d |
| --- | --- | --- | --- | --- |
| npoints0, median (IQR) | 817 (735–943) | 879 (768–1036) | 0.41 | 0.18 |
| lifetime0, median (IQR) | 0.07 (0.06–0.08) | 0.07 (0.05–0.07) | 0.10 | 0.50 |
| centroidx0, median (IQR) | 1.12 (0.96–1.26) | 1.16 (1.00–1.28) | 0.67 | 0.11 |
| centroidy0, median (IQR) | 1.69 (1.56–1.77) | 1.65 (1.59–1.76) | 0.92 | 0.062 |
| area0, median (IQR) | 1.94 (1.69–2.40) | 1.82 (1.50–2.21) | 0.17 | 0.41 |
| perimeter0, median (IQR) | 7.53 (6.91–8.19) | 7.15 (6.73–7.80) | 0.081 | 0.50 |
| filamentarity0, median (IQR) | 0.37 (0.35–0.41) | 0.37 (0.34–0.40) | 0.47 | 0.12 |
| npoints1, median (IQR) | 905 (805–1049) | 967 (836–1110) | 0.43 | 0.18 |
| lifetime1, median (IQR) | 0.07 (0.06–0.07) | 0.06 (0.05–0.07) | 0.17 | 0.38 |
| centroidx1, median (IQR) | 2.05 (1.95–2.23) | 2.04 (1.94–2.22) | 0.96 | 0.020 |
| centroidy1, median (IQR) | 2.40 (2.26–2.62) | 2.38 (2.27–2.59) | 0.59 | 0.20 |
| area1, median (IQR) | 1.32 (0.99–1.86) | 1.07 (0.75–1.39) | 0.032 | 0.57 |
| perimeter1, median (IQR) | 7.21 (5.62–8.60) | 6.43 (4.92–7.70) | 0.046 | 0.55 |
| filamentarity1, median (IQR) | 0.50 (0.44–0.56) | 0.48 (0.39–0.56) | 0.49 | 0.13 |

perimeter0, arc length of the 95% convex peels of the components; perimeter1, arc length of the 95% convex peels of the holes; area0, area of the 95% convex peels of the components; area1, area of the 95% convex peels of the holes; filamentarity0, filamentarity of the 95% convex peels of the components; filamentarity1, filamentarity of the 95% convex peels of the holes; centroidx0, x coordinate of the centroid of the 95% convex peels of the components; centroidy0, y coordinate of the centroid of the 95% convex peels of the components; centroidx1, x coordinate of the centroid of the 95% convex peels of the holes; centroidy1, y coordinate of the centroid of the 95% convex peels of the holes; npoints0, number of points of the persistence diagram for the components; npoints1, number of points of the persistence diagram for the holes; lifetime0, lifetime of the components; lifetime1, lifetime of the holes; IQR, interquartile range; NPSLE, neuropsychiatric systemic lupus erythematosus
