# Supplementary material for: Novel imaging diagnosis of neuropsychiatric systemic lupus erythematosus using topological data analysis: A retrospective study
Source: PLoS One. 2025 Aug 13;20(8):e0329859. doi: 10.1371/journal.pone.0329859 (PMC12349068; doi:10.1371/journal.pone.0329859)
Supplement: S3 Table — (DOCX) [file pone.0329859.s006.docx]

**S3 Table.** **Multiple logistic regression analysis incorporating the perimeter of the holes, history of cerebrovascular disease, age, 50% hemolytic unit of complement (CH50) levels, disease duration and prednisolone for internal use**

|  | Odds ratio | 95% CI | p-value |
| --- | --- | --- | --- |
| Perimeter1 | 1.70 | 1.03–2.80 | 0.037 |
| Cerebrovascular disease | 9.86 | 1.36–71.7 | 0.024 |
| Age | 0.94 | 0.88–1.00 | 0.037 |
| CH50 | 1.07 | 1.00–1.15 | 0.048 |
| Disease duration | 1.06 | 0.98–1.15 | 0.15 |
| Prednisolone for internal use | 1.02 | 0.97–1.09 | 0.41 |

CH50, 50% hemolytic unit of complement; CI, confidence interval; perimeter1, the arc length of 95% convex peels of holes
