# Supplementary material for: Novel imaging diagnosis of neuropsychiatric systemic lupus erythematosus using topological data analysis: A retrospective study
Source: PLoS One. 2025 Aug 13;20(8):e0329859. doi: 10.1371/journal.pone.0329859 (PMC12349068; doi:10.1371/journal.pone.0329859)
Supplement: S4 Table — (DOCX) [file pone.0329859.s007.docx]

**S4 Table. Multiple logistic regression analysis incorporating the area of the holes, history of cerebrovascular disease, age, 50% hemolytic unit of complement (CH50) levels, disease duration and prednisolone for internal use**

|  | Odds ratio | 95% CI | p-value |
| --- | --- | --- | --- |
| Area1 | 4.59 | 1.13–18.6 | 0.033 |
| Cerebrovascular disease | 11.8 | 1.46–94.9 | 0.020 |
| Age | 0.94 | 0.88–1.00 | 0.054 |
| CH50 | 1.08 | 1.00–1.16 | 0.047 |
| Disease duration | 1.07 | 0.98–1.16 | 0.13 |
| Prednisolone for internal use | 1.02 | 0.96–1.08 | 0.59 |

CH50, 50% hemolytic unit of complement; CI, confidence interval; area1, the area of 95% convex peels of the holes
