# Supplementary material for: Novel imaging diagnosis of neuropsychiatric systemic lupus erythematosus using topological data analysis: A retrospective study
Source: PLoS One. 2025 Aug 13;20(8):e0329859. doi: 10.1371/journal.pone.0329859 (PMC12349068; doi:10.1371/journal.pone.0329859)
Supplement: S5 Table — (DOCX) [file pone.0329859.s008.docx]

**S5 Table. Multiple logistic regression analysis incorporating the perimeter of the holes, age, 50% hemolytic unit of complement (CH50) levels**

|  | Odds ratio | 95% CI | p-value |
| --- | --- | --- | --- |
| Perimeter1 | 1.77 | 1.20–2.90 | 0.0099 |
| Age | 0.94 | 0.89–0.99 | 0.022 |
| CH50 | 1.06 | 1.01–1.13 | 0.037 |

CH50, 50% hemolytic unit of complement; CI, confidence interval; perimeter1, the arc length of 95% convex peels of holes
