# Supplementary material for: Novel imaging diagnosis of neuropsychiatric systemic lupus erythematosus using topological data analysis: A retrospective study
Source: PLoS One. 2025 Aug 13;20(8):e0329859. doi: 10.1371/journal.pone.0329859 (PMC12349068; doi:10.1371/journal.pone.0329859)
Supplement: S6 Table — (DOCX) [file pone.0329859.s009.docx]

**S6 Table. Multiple logistic regression analysis incorporating the area of the holes, age, 50% hemolytic unit of complement (CH50) levels**

|  | Odds ratio | 95% CI | p-value |
| --- | --- | --- | --- |
| Area1 | 4.91 | 1.60–20.0 | 0.012 |
| Age | 0.95 | 0.90–1.00 | 0.041 |
| CH50 | 1.07 | 1.01–1.14 | 0.033 |

CH50, 50% hemolytic unit of complement; CI, confidence interval; area1, the area of 95% convex peels of the holes
